# PAK Kinases Target Sortilin and Modulate Its Sorting

**DOI:** 10.1128/MCB.00411-19

**Published:** 2020-01-16

**Authors:** Lone Tjener Pallesen, Camilla Gustafsen, Jacob Flyvholm Cramer, Steen Vang Petersen, Søren Skou Thirup, Peder Madsen, Claus Munck Petersen

**Affiliations:** a The Lundbeck Foundation Research Center MIND, Department of Biomedicine, Aarhus University, Aarhus C, Denmark; b Department of Molecular Biology and Genetics, Aarhus University, Aarhus C, Denmark; c Department of Biomedicine, Aarhus University, Aarhus C, Denmark

**Keywords:** sortilin, PAK kinases, sorting, protein phosphorylation

## Abstract

The multifunctional type 1 receptor sortilin is involved in endocytosis and intracellular transport of ligands. The short intracellular domain of sortilin binds several cytoplasmic adaptor proteins (e.g., the AP-1 complex and GGA1 to -3), most of which target two well-defined motifs: a C-terminal acidic cluster dileucine motif and a YXXΦ motif in the proximal third of the domain. Both motifs contribute to endocytosis as well as Golgi-endosome trafficking of sortilin. The C-terminal acidic cluster harbors a serine residue, which is subject to phosphorylation by casein kinase. Phosphorylation of this serine residue is known to modulate adaptor binding to sortilin. Here, we show that the cytoplasmic domain of sortilin also engages Rac-p21-activated kinases 1 to 3 (PAK1-3) via a binding segment that includes a tyrosine-based motif, also encompassing a serine residue. We further demonstrate that PAK1-3 specifically phosphorylate this serine residue and that this phosphorylation alters the affinity for AP-1 binding and consequently changes the intracellular localization of sortilin as a result of modulated trafficking. Our findings suggest that trafficking of ligands bound to sortilin is in part regulated by group A PAK kinases, which are downstream effectors of Rho GTPases and are known to affect a variety of processes by remodeling the cytoskeleton and by promoting gene transcription and cell survival.

## Abstract (precis)

The multifunctional type 1 receptor sortilin is involved in endocytosis and intracellular transport of ligands. The short intracellular domain of sortilin binds several cytoplasmic adaptor proteins (e.g., the AP-1 complex and GGA1 to -3), most of which target two well-defined motifs: a C-terminal acidic cluster dileucine motif and a YXXΦ motif in the proximal third of the domain. Both motifs contribute to endocytosis as well as Golgi-endosome trafficking of sortilin.

## INTRODUCTION

Sortilin is a multifunctional type 1 receptor that is highly expressed in the nervous system but also in other tissues, including liver, kidney, spleen, and testes, and in a variety of cell types, including neurons, hepatocytes, striated muscle cells, podocytes, and mononuclear lymphocytes (1–4). Sortilin represents one of five membrane proteins that constitute the mammalian Vps10p domain receptor family, the so-called sortilins. The family members all carry an N-terminal Vps10p domain (Vps10p-D) named after the Vps10 protein, which mediates the transport of carboxypeptidase Y from the biosynthetic pathway to the vacuole in Saccharomyces cerevisiae (5). In contrast to the other family members, the luminal part of sortilin consists of only the Vps10p-D (6–9). Sortilin binds a diversity of both circulating and transmembrane targets such as nerve growth factors, neuropeptides, cytokines, and enzymes (10–17). Accordingly, it has been reported to partake in mechanisms governing neuronal death and survival, lipid metabolism, and cytokine signaling in addition to endocytosis of ligands (10, 14, 18–20). Although most of these events take place at the plasma membrane, the vast majority of sortilin is localized to the intracellular space, primarily paranuclear compartments, and engaged in trafficking between the *trans*-Golgi network, endosomes, lysosomes, and less-well-defined transport vesicles (21, 22). Yet apart from targeting internalized ligands for lysosomal degradation, the functional role(s) of sortilin trafficking is still unclear, but several studies have suggested that sortilin trafficking may facilitate protein secretion, the guided transport of other receptors, and even participation in the sorting of newly synthesized cargo proteins, e.g., proteolytic enzymes, from the *trans*-Golgi network to lysosomes (4, 17, 23, 24).

Trafficking of sortilin is controlled by interactions between its C-terminal tail and cytoplasmic adaptor proteins. Its cytoplasmic domain is short (53 amino acids) but contains binding sites for several adaptors, including adaptor protein complex 1 (AP-1) and AP-2; Golgi apparatus-localized, gamma ear-containing, ARF-binding proteins (GGAs) (GGA1 to -3 [GGA1-3]); elements of the retromer complex; Ras-related protein (Rab7b); and phosphofurin acidic cluster-sorting protein 1 (PACS-1) (20–22, 25–27). In regard to sortilin trafficking, the role of PACS-1 (and, to some extent, that of the GGAs) is still unclear, but several studies have established that AP-2 is involved in the endocytosis of sortilin, whereas the remaining adaptors participate in its Golgi-endosome cycling (21, 26, 27). A single functional site (F^787^LV^789^), possibly part of an as-yet-unidentified bipartite retromer-binding site, for retromer binding has been reported (26, 28), but two of the most important sorting sites in sortilin are particularly well defined. The first, which contributes to Golgi-endosome trafficking (and more modestly to endocytosis) and is made up of a C-terminal acidic cluster combined with a dileucine (D^823^DSDEDLL^830^), is targeted by PACS-1, AP-1 and -2, as well as the GGA proteins (20, 21). The sequence of this motif also harbors a casein kinase phosphorylation site (Ser^825^), and phosphorylation is known to modulate adaptor binding (29, 30). The second site (Y^792^SVL^795^) is a classical tyrosine-based motif (AP-1 and -2 binding) positioned in the upper one-third of the cytoplasmic domain. This motif constitutes the main site for endocytosis (AP-2) but is also of major importance for Golgi-endosome transport (AP-1) (21). Notably, the tyrosine-based motif also contains a serine residue, i.e., a potential site for phosphorylation and modulation of adaptor protein binding. In the present study, we have identified this motif as part of a site for binding of p21-activated kinases 1 to 3 (PAK1-3) and examined the functional implications of this interaction.

**FIG 1:**
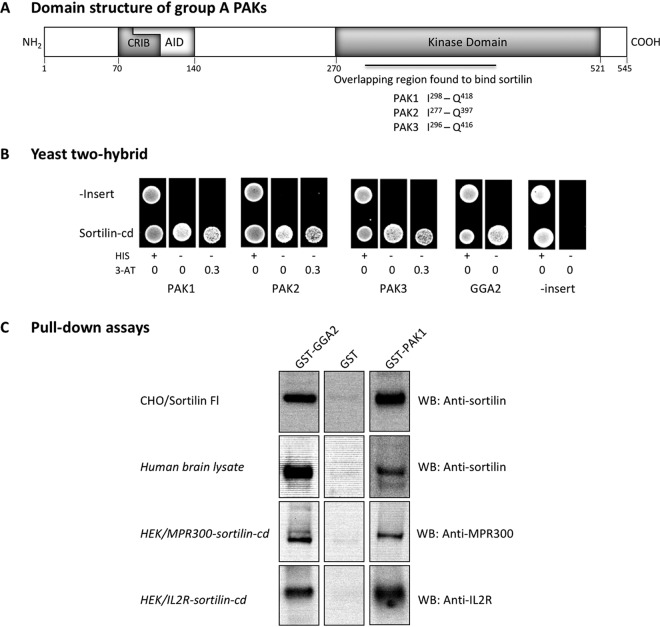
Interaction of the kinase domain of PAK1-3 with the cytoplasmic domain of sortilin. (A) Domain structure of group A PAKs. The amino acid numbering is according to human PAK1 (GenBank accession number NP_002567.3). The PAK1-3 kinases contain an N-terminal GTPase-binding domain with a Cdc42/Rac-interactive binding (CRIB) motif partly overlapped by an autoinhibitory domain (AID) and a separate kinase domain in the C terminus. The region in the kinase domain found to bind sortilin for all three kinases is marked. (B) Confirmation of interaction between sortilin and PAK kinases by yeast two-hybrid analysis. The cytoplasmic domain of human sortilin (sortilin-cd) was cloned into the Hybrigenics bait vector and tested against prey plasmids encoding human PAK1-3 or GGA2, including PAK1-T^271^-H^545^, PAK2-K^146^-Q^397^, PAK3-I^317^-R^565^, and GGA2-S^37^-N^202^. Growing colonies indicate interactions between expressed proteins. His, histidine; 3-AT, 3-amino-1,2,4-triazole. (C) Pulldown assays with GST-tagged PAK1 kinase. Full-length sortilin was pulled down from lysates of CHO/Sortilin cells and human brain and detected by Western blotting using antibodies against the sortilin ectodomain. Chimeric receptors combining the sortilin-cd with the ectodomain of either the mannose-6-phosphate receptor (MPR300–sortilin-cd) or the interleukin-2 receptor (IL-2R–sortilin-cd) were precipitated from HEK cell transfectants and detected using anti-MPR300 and anti-IL-2R antibodies, respectively. GGA2 and GST served as positive and negative controls.

The PAK family consists of six members that are all expressed in brain but otherwise differ in terms of tissue expression patterns. Based on sequence and molecular function, the family is divided into two groups: group A, consisting of PAK1-3, and group B, including PAK4-6. The group A PAKs show extensive sequence similarity, share several conserved motifs, and have similar molecular functions (31, 32). The monomers of each group A member contain an N-terminal GTPase-binding domain with a Cdc42/Rac-interactive binding (CRIB) motif partly overlapped by an autoinhibitory domain (AID) and a separate kinase domain in the C-terminal half of the molecule (Fig. 1A). Inactive kinases are homodimers in which the monomers are positioned “head to toe” and stabilized by the AIDs, which bind and inhibit the kinase domain of the opposing monomer. Following binding of a Rho GTPase (Cdc42/Rac), this interaction is disrupted, the dimer dissociates, and the free monomers are subsequently converted to active kinases by autophosphorylation.

The activated PAKs exhibit a diversity of functions reflecting their role as Cdc42 and Rac effectors. In general terms, they influence the growth, shape, and motility of neuronal as well as nonneuronal cells (for a review, see reference 33). More specifically, their activity affects the actin cytoskeleton, microtubule dynamics, membrane trafficking, neuronal connectivity, axon guidance, dendritic spine formation, and synaptic plasticity, i.e., functions that may affect or implicate Vps10p-D receptors, including sortilin.

Here, we show for the first time that PAK1-3 have a direct impact on sortilin and that each of the three group A kinases binds and phosphorylates a serine residue positioned in a sorting motif within the sortilin cytoplasmic domain. We further demonstrate that phosphorylation of sortilin reduces its adaptor protein affinity and alters its intracellular trafficking. Our findings provide new insight into the mechanisms that regulate sortilin-mediated sorting and add a new aspect to the functional repertoire of PAK1-3.

## RESULTS

The p21-activated kinases PAK1-3 were identified as potential sortilin adaptor proteins in a yeast two-hybrid (Y2H) screen using a human brain library of fragmented cDNA and the human sortilin cytoplasmic domain (sortilin-cd) as bait. Several hits were obtained with clones expressing any one of the three kinases. The cDNA expressed by the positive clones differed but represented overlapping fragments of the respective PAK kinase domains (Fig. 1A). The overlapping segments all comprised a 121-amino-acid-residue segment with almost complete (91 to 99%) sequence identity (residues I^298^ to Q^418^ of human PAK1; GenBank accession number NP_002567.3), indicating that this region is responsible for the sortilin-cd interaction. The interaction was subsequently confirmed by a positive response in Y2H tests using the sortilin-cd and the original library plasmids purchased from Hybrigenics encompassing the C-terminal kinase domains of PAK1-3 containing this 121-amino-acid-residue segment (Fig. 1B). Negative-control plasmids were included to verify that the PAK plasmids do not exhibit autoreactivity.

### The kinase domains of PAK1-3 target the sortilin-cd.

To establish the PAK-sortilin interaction at the protein level, a PAK1 fragment (Y^270^-H^545^) containing the entire kinase domain segment (Fig. 1A) was expressed in Escherichia coli as an N-terminally glutathione *S*-transferase (GST)-tagged protein. The resulting fusion protein was purified, immobilized on glutathione-Sepharose, and tested for its ability to mediate the pulldown of full-length sortilin and chimeric receptors carrying the sortilin-cd from cell and tissue lysates (Fig. 1C). Pulldown experiments using Sepharose resin coated with GST only (negative control) or GST-GGA2 (positive control) were performed in parallel (34). As apparent from Fig. 1C, Western blot (WB) analysis of the respective precipitates demonstrated that the GST-PAK1 kinase domain conveyed efficient pulldown (comparable to that of GST-GGA2) of full-length sortilin from CHO cell transfectants as well as of endogenous sortilin from human brain lysates. Moreover, the fact that chimeric receptors, combining the sortilin-cd with alternative luminal receptor domains (IL-2R [interleukin-2 receptor]–sortilin-cd and MPR300 [cation-independent mannose-6-phosphate receptor]–sortilin-cd) (21), were precipitated with similar efficiencies demonstrates that the cytoplasmic, and not the luminal, domain is responsible for the interaction with PAK1.

### The affinity of PAKs for sortilin is similar to that of established adaptor proteins.

**FIG 2:**
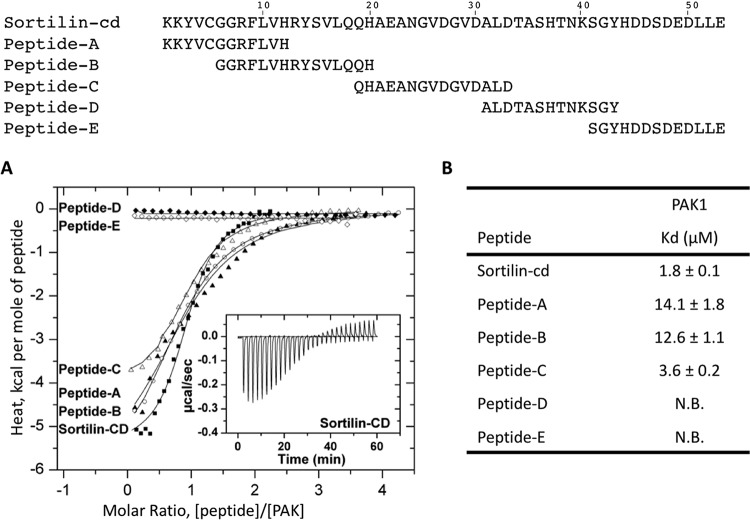
Isothermal titration calorimetry analysis of PAK1 kinase binding to the cytoplasmic domain of sortilin (sortilin-cd) or peptides thereof. (A) Graphs showing integrated heat pulses, normalized per mole of peptide injected as a function of the molar ratio (peptide/PAK1 concentration). These binding curves were fitted to the one set of sites model. The calorimetric cell contained PAK1 (25 μM), and the injection syringe contained either 100 μM sortilin-cd or peptides A to E. Squares, sortilin-cd; filled triangles, peptide A; circles, peptide B; empty triangles, peptide C; filled diamonds, peptide D; empty diamonds, peptide E. The inset graph shows raw data for the heat pulses resulting from titration of 100 μM sortilin-cd in 25 μM PAK1 in 28 injections. The trace is shown after subtraction of the heat of dilution of the peptide in buffer. (B) Dissociation constants (*K_d_*) for the different peptide-PAK1 interactions expressed as means ± standard deviations. N.B., no binding.

**FIG 3:**
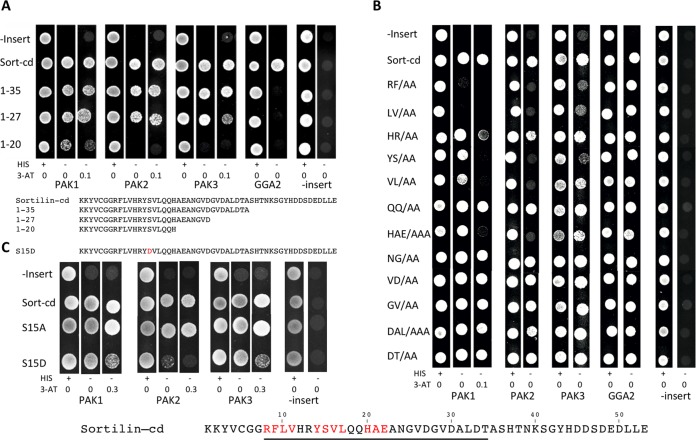
Localization of the PAK-binding site(s) in sortilin by yeast two-hybrid (Y2H) analysis. (A) Localization of the PAK-binding site in sortilin by Y2H analysis using the entire cytoplasmic domain (sort-cd) or C-terminally truncated constructs (1–35, 1–27, or 1–20) against the C-terminal kinase domains of PAK1-3. GGA2 is included as a positive control, and plasmids without inserts are used as negative controls. Growing colonies indicate interactions between expressed proteins. (B) Alanine scanning for PAK interaction sites in sortilin by Y2H analysis using a series of sortilin mutants in which two to three consecutive residues were replaced with alanine residues. Taken together, the mutations cover residues 8 to 34 of the sortilin-cd, as indicated. Amino acid residues marked in red affect binding in Y2H assays. (C) Y2H analysis investigating the effect on PAK1-3 binding when Ser^15^ in the sortilin-cd is replaced with either Ala (S15A) or Asp (S15D) (mimicking phosphorylation). His, histidine; 3-AT, 3-amino-1,2,4-triazole.

To explore the kinetics of the interaction, we next examined sortilin-kinase binding by isothermal titration calorimetry (ITC). The complete cytoplasmic domain of sortilin was expressed as a GST-tagged protein and purified prior to the removal of the GST tag by enzymatic cleavage with thrombin. Titration of 25 μM GST-PAK with 100 μM sortilin-cd was performed in a buffer containing 100 mM NaCl and 50 mM Tris-HCl at pH 7.4. The analysis showed a dissociation constant (*K_d_*) of ∼2 μM (Fig. 2), reflecting an affinity similar to that of the interaction between sortilin and GGA2 (30). Corresponding ITC analyses of the interactions between PAK1 and five partly overlapping peptides (peptides A to E, each comprising 12 to 15 amino acid residues) covering the entire sequence of the sortilin-cd further indicated that the responsible binding site(s) was located in the N-terminal part of the sortilin-cd (Fig. 2). In agreement with this conclusion, a Y2H test of the sortilin-cd and three C-terminally truncated cytoplasmic domain constructs demonstrated that the binding site(s) for PAK1 resides within the first 27 residues of the cytoplasmic domain and the removal of the last (C-terminal) 26 amino acid residues have little or no effect on the interaction (Fig. 3A).

### The PAK-binding segment of sortilin includes a functional sorting motif.

Based on these data, we next set up a Y2H screen of wild-type (wt) and mutant sortilin-cd constructs in order to identify the residues involved in PAK binding. Positive PAK cDNA clones (the translated products are shown in Fig. 1A) were tested against a series of sortilin mutants in which two or three consecutive residues were replaced with alanine. Taken together, the mutations cover the sequence from R^786^ to T^812^ (residues 8 to 34 of the sortilin-cd), i.e., close to the collected sequences of the binding-active sortilin-cd peptides A, B, and C (Fig. 2) (below, the residues are simply designated residues 1 to 53 according to their position in the cytoplasmic domain). As displayed in Fig. 3B, the outcome of the alanine-scanning experiment identified the cytoplasmic domain sequence R^8^FLV^11^ as a key determinant in the binding of all three PAKs and further suggested an additional and significant contribution by Y^14^SVL^17^ and H^20^AE^22^. In other words, the residues involved in the binding of the PAK A kinase domains encompass a key adaptor-binding site (YSVL) and, notably, a sequence that complies with the basic requirements for phosphorylation of Ser^15^ by PAK.

Interestingly, an additional Y2H screen (Fig. 3C) revealed that whereas the replacement of Ser^15^ with Ala had no effect on PAK1-3 binding, an Asp-for-Ser substitution (imitating phosphorylation) caused significantly weaker signals (in particular with regard to PAK2). Thus, the sortilin tail appears to lose its affinity for all three PAKs upon the phosphorylation of Ser^15^. The Y2H analysis further reveals that peptides A, B, and C (Fig. 2) all comprise parts of the, but not the complete, binding sequence, which explains their lower affinities for PAK than the full-length cytoplasmic domain and furthermore why the nonoverlapping peptides A and C independently confer binding to PAK.

### PAKs phosphorylate the sortilin-cd.

**FIG 4:**
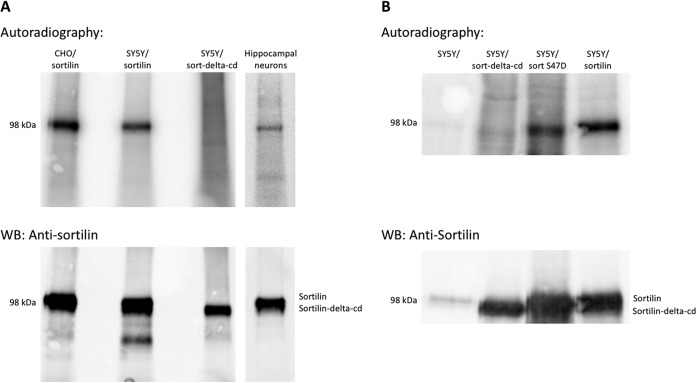
Phosphorylation of sortilin in cell lines and primary cultures. (A) Phosphorylation of sortilin was investigated by ^32^P labeling primary cultures of murine hippocampal neurons as well as CHO and SY5Y cells stably transfected with sortilin and subsequently immunoprecipitating sortilin from cell lysates. Cells transfected with a sortilin deletion mutant lacking the cytoplasmic domain (sortilin-delta-cd) were used as negative controls. (Top) Immunoprecipitated proteins were separated by SDS-PAGE and electroblotted onto a PVDF membrane, and phosphorylation was analyzed by autoradiography. (Bottom) Immunoprecipitated sortilin was identified by Western blotting of the membrane using mouse antisortilin (catalog number 612100; BD Biosciences). (B) ^32^P labeling of untransfected SY5Y cells and cells transfected with either full-length sortilin, sortilin-delta-cd, or a sortilin S47D mutant. Cell labeling and analysis of immunoprecipitated proteins were performed as described above.

It is well known that Ser^47^ in the C-terminal GGA-binding sequence of sortilin is phosphorylated *in vivo* by casein kinase and that this serves to modulate adaptor binding and sorting of the receptor. To confirm previous findings and to pursue the possibility of alternative phosphorylation sites in the sortilin-cd, we therefore performed ^32^P labeling of CHO and SY5Y cells stably transfected with wt sortilin or, as a negative control, a sortilin mutant lacking the cytoplasmic domain (sortilin-delta-cd). The cells were incubated with carrier-free ^32^P-labeled *o*-phosphate in phosphate-free Dulbecco’s modified Eagle’s medium (DMEM) for 4 h prior to 1 h of incubation in the presence of the phosphatase inhibitor calyculin. The cells were then lysed in a 1% Triton X-100 buffer supplemented with phosphatase and proteinase inhibitors, and sortilin was immunoprecipitated from the lysates using antibodies directed against the receptor ectodomain. Analysis by SDS-PAGE and autoradiography established that sortilin was indeed phosphorylated and that the specific site of phosphorylation was localized to the cytoplasmic domain (Fig. 4A). Similar results were obtained in ^32^P-labeled primary cultures of wt hippocampal mouse neurons (expressing endogenous levels of sortilin) (Fig. 4A, lane 4).

To clarify whether Ser^47^ was responsible for all the observed labeling or if other sites contributed, we next compared ^32^P labeling of sortilin in SY5Y cells transfected with either wt sortilin or a mutant receptor in which Ser^47^ was replaced with an Asp residue. Cell labeling and analysis of immunoprecipitated proteins were performed as described above, and the two transfectants were examined in parallel (Fig. 4B). The results showed that the phosphorylation of sortilin persisted despite the absence of Ser^47^, and based on a comparison between the degrees of labeling in the two constructs (radioactivity relative to the amount of protein precipitated), alternative sites appear to be responsible for about 50% of the observed phosphorylation of wt sortilin.

### PAKs specifically phosphorylate Ser^15^ of the sortilin-cd and alter its trafficking.

**FIG 5:**
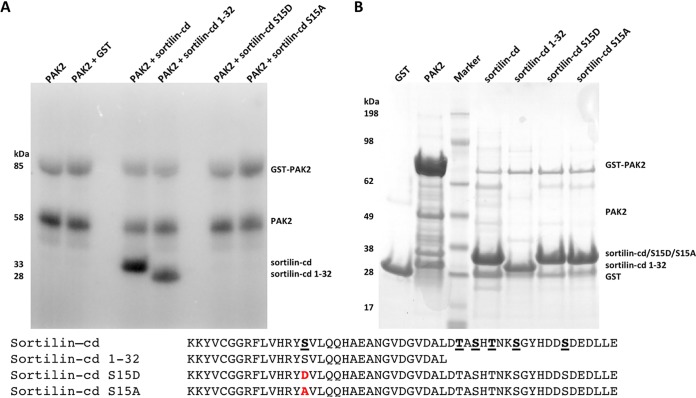
*In vitro* phosphorylation of sortilin by group A PAK kinases. (A) *In vitro* phosphorylation of sortilin with a constitutively active PAK2 kinase. GST-tagged constructs of the sortilin cytoplasmic domain were expressed and purified, including the sortilin-cd, a cytoplasmic domain deletion mutant (sortilin-cd 1–32) containing only a single potential Ser phosphorylation site, and two single point mutants of Ser15 in the sortilin-cd, sortilin-cd S15A and sortilin-cd S15D. The *in vitro* phosphorylation assay was performed using ^32^P-labeled ATP, full-length GST-PAK2 T402E, and the purified fusion protein or GST as a negative control. The phosphorylation reaction was stopped after 30 min and analyzed by SDS-PAGE and subsequently autoradiography. (B) Evaluation of all isolated GST-tagged fusion proteins used for the *in vitro* phosphorylation assay analyzed by SDS-PAGE and Coomassie brilliant blue staining. Amino acid sequences of the sortilin cytoplasmic domain are shown, with the potential Ser and Thr phosphorylation sites underlined. Amino acid residues mutated in sortilin-cd S15D/A are marked in red.

Mass spectrometry (MS) analysis of phosphopeptides recovered from a tryptic digest of the *in vitro*-phosphorylated sortilin-cd demonstrated the presence of a single phosphorylation site encompassed in the Y^14^-K^40^ tryptic fragment, which includes the potential PAK target (RY)S^15^. Our data suggest that the cytoplasmic domain of sortilin indeed encompasses a PAK-specific site of phosphorylation, but the tryptic peptide contains three additional Ser or Thr amino acid residues, and we were not able to assign the specific site of phosphorylation by mass spectrometry. However, *in vivo* phosphorylation of Ser^15^ was recently reported (35), and to determine if the sortilin-cd, and Ser^15^ in particular, may in fact be subject to PAK kinase activity, we tested PAK’s capacity for *in vitro* phosphorylation of the wt sortilin-cd and selected mutant constructs of the cytoplasmic domain (Fig. 5). The receptor constructs were generated with an N-terminal GST tag, expressed in E. coli, and purified from culture medium by glutathione affinity chromatography. Each of the purified sortilin-cd variants was then incubated with constitutively active PAK2 (T402E) in the presence of [γ-^32^P]ATP. After 30 min, the reaction was stopped, and the samples were examined by SDS-PAGE and autoradiography. As apparent from Fig. 5, wt sortilin and a deletion mutant comprising only Ser^15^ (sortilin-cd 1–32) was readily phosphorylated, whereas mutants carrying a single substitution at position 15 (Asp or Ala for Ser) showed no trace of radioactivity.

It can be concluded that PAK1-3 may indeed instigate the phosphorylation of sortilin and that they target a single serine residue (Ser^15^) located in the kinase domain-binding site of the sortilin-cd.

### Phosphorylation of Ser^15^ in the sortilin-cd alters its trafficking.

**FIG 6:**
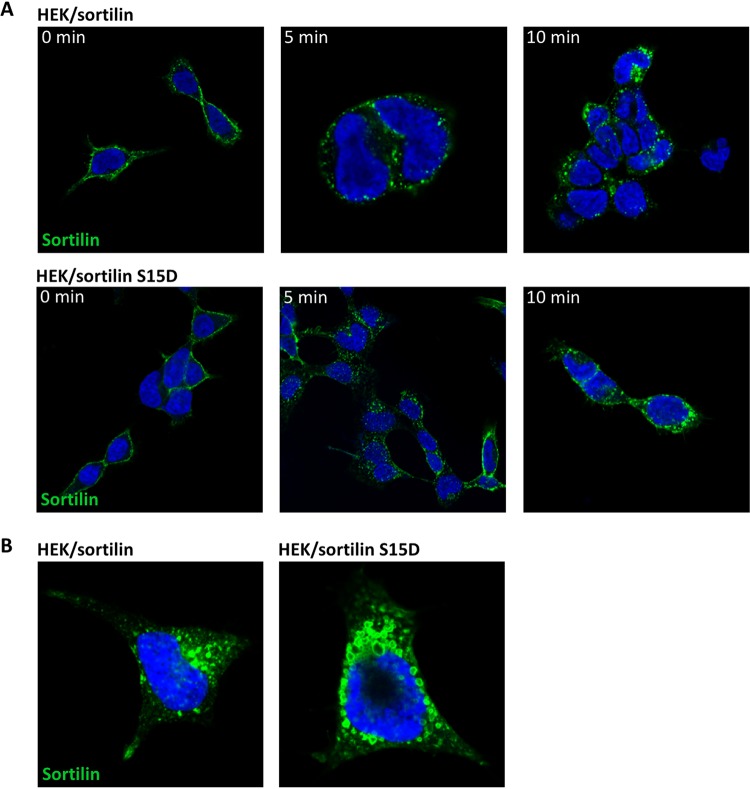
Cellular internalization and localization of the sortilin S15D mutant mimicking constitutive PAK phosphorylation. (A) Cellular internalization. HEK cells stably expressing the wild-type or an S15D mutated sortilin receptor were incubated for 1 h on ice with monoclonal mouse antisortilin clone F11 antibodies. Cells were washed; incubated at 37°C for 0, 5, or 10 min; and finally fixed in 4% paraformaldehyde and permeabilized. Following fixation, cells were stained with Alexa Fluor 488-conjugated donkey anti-mouse antibody (green), and the nuclei were visualized by using Hoechst stain (blue). (B) Immunocytochemistry of sortilin in transfected HEK cells showing the localization of wt sortilin and sortilin S15D. Cells were stained with monoclonal mouse antisortilin clone F11 antibody (green), and nuclei were visualized with Hoechst stain (blue).

Given that Ser^15^ is part of the short signal sequence which governs endocytosis and, to a large extent, also Golgi-endosome sorting of sortilin, we decided to examine if the phosphorylation of Ser^15^ impacts sortilin trafficking. To this end, we first compared the internalization of wt sortilin to that of a mutant receptor in which Ser^15^ had been replaced with an Asp residue to mimic permanent phosphorylation. HEK cells expressing either the wt or the mutant receptor were incubated for 1 h with antisortilin antibodies at 4°C and then washed in unsupplemented warm medium. After a maximum of 40 min at 37°C, the cells were fixed and stained with a fluorescent secondary antibody, and the degree of receptor endocytosis was determined by fluorescence microscopy. The results (Fig. 6A) revealed the almost complete internalization of both receptors, signifying little or no difference between their capacities for endocytosis.

However, the detection (immunofluorescence) of the two receptors at steady state (Fig. 6B) suggested that mutant sortilin (sortilin S15D) was more widely distributed in the cells than wt sortilin, which was mainly concentrated in paranuclear compartments. Also, sortilin S15D-associated compartments included large vesicular structures that were not seen upon staining for wt sortilin.

**FIG 7:**
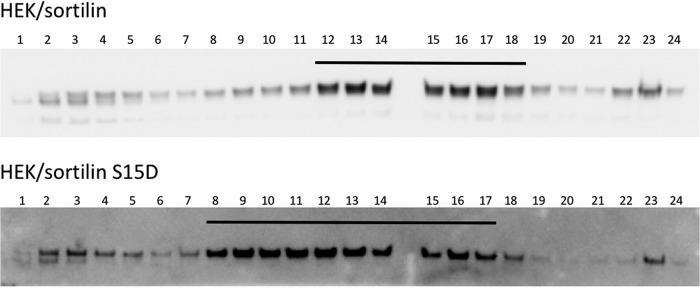
Effect of phosphorylation on the subcellular localization of sortilin. Subcellular fractionation of HEK cells transfected with either wt sortilin or S15D mutated sortilin (HEK/sortilin and HEK/sortilin S15D cells, respectively) was performed by centrifugation in a discontinuous iodixanol density gradient. Twenty-four fractions were collected by ultracentrifugation and subsequently analyzed by Western blotting using mouse antisortilin (catalog number 612100; BD Biosciences). Bars indicate the distribution of fractions with the largest amount of sortilin.

The overall cellular localization of the receptors (reflecting trafficking) was therefore next determined by subcellular fractionation. Cells expressing either wild-type or mutant (S15D) sortilin were examined in parallel, and as apparent by subsequent WB of the fractionated samples, the distributions of the two receptors clearly differed (Fig. 7). Similar results were obtained in two separate but identical experiments.

Despite the difference in localization/trafficking, the sortilin S15D mutation did not appear to affect the turnover of sortilin. Thus, in pulse-chase experiments with biolabeled receptors, wild-type sortilin and the mutant receptor displayed similar half-lives (not shown).

### Phosphorylation (Ser^15^) downregulates the sortilin–AP-1 interaction.

**FIG 8:**
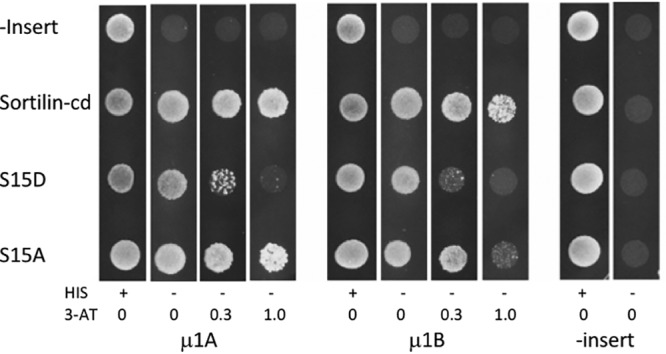
Phosphorylation of sortilin inhibits its interaction with AP-1. Yeast two-hybrid (Y2H) analysis investigating the effect of phosphorylation of Ser^15^ in the cytoplasmic domain of sortilin on the interaction with the μ1A and μ1B subunits of AP-1. Y2H analysis was performed by using the entire cytoplasmic domain of sortilin (sortilin-cd), sortilin S15D, or sortilin S15A against the μ1A and μ1B subunits of AP-1. Plasmids without inserts were included as negative controls. Growing colonies indicate interactions between expressed proteins. His, histidine; 3-AT, 3-amino-1,2,4-triazole.

Since the S15D mutation did not significantly affect the endocytosis or turnover of sortilin, we deduce that the observed difference in subcellular distribution must result from a change in intracellular trafficking induced by the point mutation mimicking phosphorylation. As mentioned above, the Y^14^XXL^17^ motif partakes in both endocytosis and the Golgi-endosome transport of sortilin. Tyr^14^ plays a dominant role in endocytosis but does not affect the AP-1-dependent Golgi-endosome transport of chimeric receptors carrying the luminal domain of MPR300 (cation-independent mannose-6-phosphate receptor) in combination with the sortilin cytoplasmic domain. In contrast, substitution of Ala for both Tyr^14^ and Leu^17^ significantly reduced the MPR300/sortilin chimera-mediated sorting of newly synthesized lysosomal enzymes, suggesting that Val^16^Leu^17^ is the key residue(s) in the latter context (21). We therefore performed a Y2H analysis to test if the phosphorylation of Ser^15^ could alter the function of the dileucine-like (Val^16^-Leu^17^) motif, i.e., its interaction with the μ1A and μ1B subunits of AP-1. The outcome is depicted in Fig. 8, which clearly demonstrates that compared to wt sortilin, the S15D mutant exhibits a distinct reduction in its ability to interact with each of the two μ1 subunits. However, the capacity for μ1 subunit interactions of another mutant, S15A, appeared to be equal to that of the wt sortilin construct, signifying that the reduced interaction between the AP-1 subunits and the S15D mutant owed itself to the introduction of a negative charge, i.e., phosphorylation or, in this case, Asp.

We conclude that the PAK1-3 kinases target and phosphorylate Ser^15^ of the sortilin-cd and thereby alter the intracellular trafficking of the receptor predominantly by reducing its affinity for AP-1, which mediates Golgi-endosome sorting of wild-type sortilin.

## DISCUSSION

Sortilin is a multifunctional sorting receptor that binds a variety of extracellular ligands, including both soluble and transmembrane proteins. It partakes in the endocytosis of ligands, but the major pool of the receptor is intracellular and engaged in trafficking between different compartments, notably between the Golgi apparatus and endosomes (21, 22). Accordingly, sortilin interacts with several cytoplasmic adaptor proteins via specific motifs in its short cytoplasmic domain.

The present study was undertaken in an attempt to identify new binding partners for the sortilin-cd, and here, we report its interaction with the p21-activated kinases PAK1-3 and the functional implications of the interaction.

### A novel motif in the sortilin-cd.

**FIG 9:**
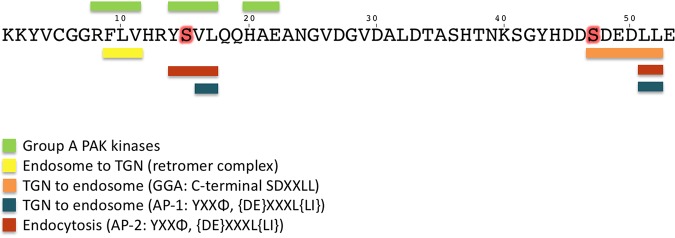
Adaptor binding and phosphorylation sites. The 53 amino acids constituting the cytoplasmic domain of sortilin and a summary of the known adaptor-binding sites, including the novel group A PAK-binding site, are shown. The extreme C terminus comprises an acidic cluster combined with a dileucine motif and constitutes a target site for binding of AP-1 and AP-2 complexes as well as GGA1-3. The upper juxtamembrane segment harbors at least two separate sites, one binding the retromer complex and the other binding AP-1 and AP-2. Our findings reveal that the two sites are furthermore part of yet another binding sequence that mediates interactions with the kinase domain of the group A PAKs. Serine residues that are targets for phosphorylation are marked in red, including the PAK phosphorylation sites Ser^15^ and Ser^47^ in the GGA-binding site. TGN, *trans*-Golgi network.

The established adaptor-binding motifs of sortilin are localized in the extreme C terminus of the cytoplasmic domain and in the upper one-third of its juxtamembrane (Fig. 9). The C-terminal site comprises an acidic cluster combined with a dileucine motif (AC-dileucine) and constitutes a target for AP-1 complexes, GGA1-3, and PACS-1 (20, 21). The role of PACS-1 is still unresolved, but AP-1, and most likely GGAs, is involved in Golgi-endosome transport (21, 29). Moreover, the dileucine contributes to endocytosis and interacts with AP-2 complexes. The juxtamembrane segment harbors at least two separate sites, i.e., a short segment (F^9^LV^11^), which may serve as a binding site for the retromer complex, and a second segment (Y^14^SVL^17^) fitting the canonical AP-1- and -2-binding motif YXXΦ (where X is any residue and Φ is a hydrophobic residue) (21, 26). Our findings now reveal that both motifs are part of yet another binding sequence forming a motif (R^8^FLV-[XX]-YSVL-[XX]-HAE^22^) that mediates interaction with the kinase domain in each of the three PAKs. Analysis of the interaction showed that sortilin and PAK coprecipitate from cells and brain tissue, and ITC measurements of binding established a *K_d_* of about 2 μM, signifying an affinity comparable to that of GGA adaptors for sortilin and confirming its functional relevance (30).

### The sortilin-cd is a PAK substrate.

Interestingly, the YSVL sorting motif, which is part of the PAK-binding sequence, also harbors a serine that appeared as a potential site for phosphorylation by PAK. It is well known that the sortilin-cd is subjected to phosphorylation *in vivo* and that casein kinase 2 mediates the phosphorylation of Ser^47^ (29, 30). However, our MS analysis of *in vivo* ^32^P-labeled sortilin demonstrates that only about 50% of the phosphorylation is accounted for by Ser^47^, and *in vitro* experiments with the purified sortilin-cd presented Ser^15^ as a functional and specific site for PAK phosphorylation. These findings are in accordance with those of a recent study by Li et al., who reported the phosphorylation of both Ser^15^ and Ser^47^ in sortilin obtained from sortilin-expressing HepG2 cells as well as from mice overexpressing sortilin (35). In concert, these data establish that PAKs can phosphorylate Ser^15^ and that Ser^15^ is a target for phosphorylation *in vitro* as well as *in vivo*.

Furthermore, Li et al. reported elevated levels of circulating lipids and (Ser^15^-)phosphorylated sortilin in obese mice compared to the levels in wild-type mice. Likewise, the level of Ser^15^-phosphorylated sortilin increased 2- to 3-fold in HepG2 cells stimulated with palmitate. As lipids have been shown to stimulate PAK kinase activity (36), our findings suggest that the observations reported by Li et al. reflect enhanced PAK-mediated phosphorylation of sortilin *in vivo*.

### Phosphorylation of Ser^15^ impacts sortilin trafficking.

In terms of the functional implications of Ser^15^ phosphorylation, however, conclusions differ. Li et al. observed a downregulation of cellular sortilin protein upon Ser^15^ phosphorylation and ascribed this to enhanced ubiquitination (at Lys^40^) and the resulting increase in sortilin degradation. In contrast, we find no significant changes between the half-life of S15D sortilin mutants and that of wild-type receptors. Instead, the present results obtained by cellular expression of S15D mutants and by Y2H analysis using a mutated sortilin-cd strongly indicate that Ser^15^ phosphorylation alters the intracellular localization of sortilin due to a lowered affinity for AP-1 and a change in sorting. As mentioned above, Ser^15^ is positioned in the YXXΦ motif (Y^14^SVL^17^), which serves in endocytosis as well as in Golgi-endosome trafficking. We previously demonstrated that both Tyr^14^ (in particular) and Leu^17^ are important for receptor endocytosis and account for about 80% of the total endocytic activity of sortilin (21). In contrast, a Y14A substitution had no apparent effect on Golgi-endosome transport, while double mutants (Y14A and L17A) presented an ∼50% reduction, indicating that the YSVL motif might in fact be a combination of two functional motifs, i.e., Y^14^XXL^17^ for endocytosis and dileucine-like V^16^L^17^ for Golgi-endosome sorting (21). The present results seem to support this notion, as the S15D mutation (i.e., phosphorylation) affected intracellular sorting and AP-1 binding without significantly affecting endocytosis. Moreover, a previous study reporting a comparable half-life of sortilin in AP-1 knockout and wild-type cells supports our finding that the phosphorylation of Ser^15^ results in a decreased affinity for AP-1 complexes and “missorting” but not in a shorter sortilin half-life (20). Recent findings suggest that retromer binding in yeast is conveyed by a bipartite site in receptor cytoplasmic domains (28). This could evidently also be the case for mammalian receptors such as sortilin. However, as the phosphorylation of Ser^15^ did not increase sortilin turnover (degradation), as might be expected upon an insufficient retrieval of the receptor, it appears that phosphorylation does not affect the retromer-binding site(s).

It follows that each of the two major adaptor-binding sites in sortilin is targeted by kinases. Ser^47^ in the C-terminal AC-dileucine motif is phosphorylated by casein kinase 2 and modulates GGA binding, and as shown here, PAK1-3 phosphorylate Ser^15^, thereby modulating AP-1 binding and the distribution of cellular sortilin. As PAKs are found in all tissues expressing sortilin and are activated via many pathways, it seems likely that they actively modulate sortilin trafficking under several conditions (36–38).

In summary, we have shown that sortilin interacts with the kinase domains of PAK1-3. The endocytic and Golgi-endosome sorting motif Y^14^SVL^17^ is part of the PAK-binding sequence, and PAKs phosphorylate Ser^15^. Upon phosphorylation, sortilin loses its affinity for AP-1 complexes as well as for PAKs and alters its cellular localization and trafficking. PAKs have numerous targets, but as far as we know, sortilin is the first example of a PAK receptor substrate. Thus, the present findings present new functional aspects of both PAKs and sortilin.

## MATERIALS AND METHODS

### DNA constructs and recombinant proteins. (i) Sortilin.

Full-length human sortilin (831 amino acid residues) (GenBank accession number NP_002950) and mutated constructs were expressed using the pcDNA3.1/Zeo(−) vector (Invitrogen). Full-length sortilins with the serine at position 793 (residue 15 in the cytoplasmic domain) (for the sequence, see Fig. 2) replaced by aspartate or alanine (sortilin S15D or S15A) and the serine at position 825 replaced by aspartate (residue 47 in the cytoplasmic domain) (sortilin S47D) were created by overlapping PCR. Sortilin lacking the cytoplasmic domain (M^1^-C^783^) (sortilin-delta-cd) was cloned into the pcDNA3.1/Zeo(−) vector. Chimeric receptors expressing the luminal and transmembrane domains of the interleukin-2 receptor (IL-2R) and the cation-independent mannose-6-phosphate receptor (MPR300) fused to the cytoplasmic tail of human sortilin (IL-2R–sortilin-cd and MPR300–sortilin-cd, respectively) were previously described (21).

### (ii) Plasmids for yeast two-hybrid assays.

Library and bait plasmids were purchased from Hybrigenics (Paris, France). The cytoplasmic domain of human sortilin (K^779^-E^831^) was cloned into the Hybrigenics bait vector pB27 in fusion with LexA. The sortilin-cd deletion mutants cd 1–35 (K^779^-A^813^), cd 1–27 (K^779^-D^805^), and cd 1–20 (K^779^-H^798^) were likewise cloned into pB27. The sortilin dialanine scan mutants and sortilin-cd S15D/A (Ser^793^) mutants were created by overlapping PCRs. The purchased pP6 prey plasmids encoding human PAK1-3 and GGA2 were sequenced, revealing the following fragments: the region of PAK1 spanning residues T^271^ to H^545^ (PAK1-T^271^-H^545^) (GenBank accession number NP_002567.3), PAK2-K^146^-Q^397^ (GenBank accession number NP_002568.2), PAK3-I^317^-R^565^ (GenBank accession number NP_001121644), and GGA2-S^37^-N^202^ (GenBank accession number NP_055859.1). The AP-1 adaptor complex subunits μ1A (GenBank accession number NP_115882.1) and μ1B (GenBank accession number NP_005489.2) inserted into the Matchmaker vector pACT2 (GenBank accession number U29899) were kind gifts from Juan S. Bonifacino (NIH, Bethesda, MD).

### (iii) GST-tagged proteins.

The following proteins were expressed as N-terminal GST-tagged fusion proteins in E. coli BL21 using the pGEX4T1 vector (GE Healthcare): the complete sortilin-cd K^779^-E^831^ (K^1^-E^53^, residues in the cytoplasmic domain), sortilin-cd S15D, sortilin-cd S15A, and a sortilin-cd deletion mutant lacking the 21 C-terminal amino acids (K^779^-L^810^). The VHS domain of GGA2 (M^1^-L^167^) (GenBank accession number NP_055859.1) and the C terminus of PAK1 (Y^270^-H^545^) (GenBank accession number NP_002567.3) corresponded to the fragment identified by the yeast two-hybrid screen. A cDNA clone, pGEX6P-1-PAK2 G258R T402E, expressing constitutively active full-length human GST-PAK2 (T402E) (DU4558) (GenBank accession number BC069613) was obtained from the MRC PPU Reagents and Services facility (University of Dundee, Scotland) (39–41).

### Transfected cell lines.

CHO-K1, HEK293, and SH-SY5Y cells were transfected using FuGENE transfection reagent (Roche). CHO cells were cultured in serum-free HyClone HyQ-CCM5 CHO cell culture medium (GE Healthcare), HEK293 cells were cultured in Dulbecco’s modified Eagle’s medium (DMEM; Bio-Whittaker) with 10% fetal bovine serum (FBS; Gibco), and SH-SY5Y cells were cultured in DMEM–F-12 medium (Bio-Whittaker) with 10% FBS. In addition, all cell culture media were supplemented with 100 U penicillin plus 100 μg/ml streptomycin (Sigma). Stably transfected clones were selected by using 100 μg/ml zeocin (Invitrogen). Positive clones were identified by immunocytochemistry. The pcDNA3.1/Zeo(−) constructs described above, containing full-length sortilin or an IL-2R–sortilin-cd chimera, were transfected into CHO cells; HEK cells were transfected with full-length sortilin, sortilin S15D, and the MPR–sortilin-cd chimera; or, finally, SY5Y cells were transfected with full-length sortilin, sortilin-delta-cd, or sortilin S47D.

### Primary cultures.

Primary cultures of hippocampal neurons from mice were prepared from wild-type C57BL/6J postnatal day 0 pups. For neuron cultures, tissue was digested in papain (20 U/ml); seeded into poly-l-lysine–laminin-coated dishes in neurobasal medium (Invitrogen) supplemented with B27 (Invitrogen), GlutaMAX (Invitrogen), Primocin (Lonza), and 20 μM 5-fluoro-2′-deoxyuridine (Sigma); and maintained at 37°C with 5% CO_2_ for 10 to 12 days.

### Western blotting.

Western blot (WB) analyses were performed using precast NuPAGE 4 to 12% Bis-Tris protein gels (Invitrogen) and polyvinylidene difluoride (PVDF) membranes, and following SDS-PAGE and electroblotting, membranes were probed with the following antibodies: antisortilin (catalog number 5264; custom-made by Dako) (43), anti-IL-2R (anti-CD25; Roche), and rabbit anti-human MPR300 (2C2; custom-made). Proteins were visualized with horseradish peroxidase (HRP)-conjugated secondary antibodies (Dako) and the ECL WB substrate (Pierce) using a Las-4000 imager (Fujifilm) and quantified using Multi Gauge v.3.2 software (Fujifilm).

### GST pulldown of sortilin from transfected cells and brain homogenates.

For pulldown experiments on human brain homogenates, 10-g frozen brain tissue samples were homogenized on ice in 10 ml lysis buffer (20 mM HEPES-KOH, 125 mM potassium acetate, 2.5 mM magnesium acetate, 320 mM sucrose, 0.1 mM EDTA [pH 7.6]) containing protease inhibitors (cOmplete mini; Roche). The homogenate was centrifuged (10 min at 1,000 × *g* at 4°C), Triton X-100 was added to a final concentration of 1.25% (vol/vol), and the mixture was vortexed and rotated for 60 min at 4°C. Soluble protein fractions were generated by centrifugation for 15 min at 20,000 × *g* at 4°C and subsequent ultracentrifugation for 60 min at 100,000 × *g* at 4°C. For GST pulldowns from transfected cell lines, CHO cells stably expressing either full-length sortilin or an IL-2R–sortilin-cd chimera and HEK293 cells transfected with MPR–sortilin-cd were lysed on ice in lysis buffer (150 mM NaCl, 2 mM MgCl_2_, 0.1 mM EGTA, 2 mM CaCl_2_, and 10 mM HEPES [pH 7.4] with cOmplete mini protease inhibitors and 1% [vol/vol] Triton X-100) and centrifuged for 10 min at 18,400 × *g* at 4°C. A total of 500 μl of the supernatant from the human brain homogenate was mixed with either 100 μg of GST, GST-GGA2, or GST-PAK1, adjusted to a volume of 10 ml with lysis buffer (without Triton X-100), and incubated with rotation overnight at 4°C. Similarly, 100 μl of the supernatant from cell lysates was mixed with 10 μg GST, GST-GGA2, or GST-PAK1, adjusted to a volume of 1 ml in lysis buffer (without Triton X-100), and rotated overnight at 4°C. The following day, 100 μl glutathione-Sepharose 4B beads was added, and samples were rotated for 4 h at 4°C. Beads were pelleted, washed four times for 5 min in lysis buffer (modified to 0.4 M NaCl, without Triton X-100), and finally eluted with 100 μl SDS-PAGE sample buffer with dithioerythritol (DTE) and analyzed by WB as described above, using rabbit antisortilin (catalog number 5264), anti-MPR (2C2), or anti-IL-2R (anti-CD25; Roche).

### Yeast two-hybrid screening.

The human sortilin cytoplasmic domain was cloned into pB27, a Y2H vector optimized by Hybrigenics, and subsequently transformed into the L40GAL4 yeast strain (42). An adult human brain random-primed cDNA library, transformed into the Y187 yeast strain, was used for mating. Following selection on medium lacking leucine, tryptophan, and histidine (−Leu/−Trp/−His), positive clones were picked, and the corresponding prey fragments were amplified by PCR and sequenced at their 5′ and 3′ junctions. To certify data from the primary screening, positive preys from the yeast-transformed cDNA library and bait plasmids were purchased from Hybrigenics for additional verification using control plasmids without inserts. Transformed cells were suspended in sterile double-distilled water (ddH_2_O) and spot plated (5 μl/spot) onto agar plates prepared from 46 g/liter yeast minimal SD agar base (Clontech) with 100 μg/ml penicillin-streptomycin and supplemented with either 640 mg/liter −Leu/−Trp dropout supplement (Clontech) for control plates or 640 mg/liter −Leu/−Trp/−His dropout supplement (Clontech) for test plates, and 3-amino-1,2,4-triazole (3-AT; Sigma-Aldrich) was added to the plates to strengthen the conditions of the interaction.

### Isothermal titration calorimetry.

Binding of sortilin-cd and peptides A to E, each comprising 12 to 15 amino acid residues (CASLO) to GST-tagged PAK1 (Y^270^ to H^545^), was measured by isothermal titration calorimetry (ITC). The complete wt sortilin-cd was expressed as a GST-tagged peptide and purified prior to the removal of the GST tag by thrombin cleavage. The titration experiments were performed on a MicroCal VP-ITC isothermal titration calorimeter (MicroCal Inc.). GST-PAK1 for ITC was prepared as described above and dialyzed against 100 mM NaCl–50 mM Tris-HCl buffer (pH 7.5). Peptides were dissolved in the same buffer, and to ensure accurate measurements, concentrations of protein and peptides were determined by analysis of total amino acids. All solutions were filtered, degassed, and equilibrated to the corresponding temperature before each experiment. In a typical ITC experiment, 1.45 ml GST-PAK1 (25 μM) was titrated at 34°C with peptides A to E (100 μM) or the sortilin-cd (100 μM) in 28 steps of 10 μl. The time between injections was set to 150 s, and the syringe mixing speed was set at 300 rpm. Heat evolving from dilution was measured by injecting the ligand into Tris-HCl buffer. This heat of dilution was subtracted from the heat of the reaction to obtain the effective heat of binding. Finally, the equilibrium dissociation constant (*K_d_*) for the binding processes was determined using ORIGIN software (OriginLab Corporation).

### Phosphorylation assay.

For ^32^P labeling of hippocampal neurons, untransfected SY5Y and cells transfected with full-length sortilin, sortilin-delta-cd, or sortilin S47D were cultured in monolayers in poly-l-lysine-coated 6-well plates. At about 90% confluence, cells were washed 3 times with 5 ml phosphate-free DMEM (catalog number 11971025; Gibco) with Na-pyruvate (Invitrogen) added. Cells were then added to fresh phosphate-free DMEM supplemented with carrier-free ^32^P-labeled orthophosphate (0.4 mCi/ml), and cells were incubated at 37°C with 5% CO_2_ for 4 h prior to an additional hour of incubation in the presence of 70 nM calyculin (Cell Signaling Technology). Cells were then washed with cold phosphate-buffered saline (PBS) buffer (HyClone Dulbecco PBS [DPBS]; Thermo Scientific) containing phosphatase inhibitors (PhosSTOP; Roche), and finally, the cells were lysed on ice with immunoprecipitation (IP) buffer (150 mM NaCl, 2 mM MgCl_2_, 0.1 mM EGTA, 2 mM CaCl_2_, and 10 mM HEPES [pH 7.4] with a cOmplete mini protease inhibitor cocktail [Roche] and PhosSTOP [Roche]) containing 1% Triton X-100. Lysates were collected from the wells, centrifuged at 18,000 × *g* for 10 min at 4°C, and diluted four times in IP buffer. Sortilin was then immunoprecipitated for 3 h at 4°C from the supernatants using GammaBind G Sepharose beads (GE Healthcare) preincubated with polyclonal rabbit antisortilin antibodies (catalog number 5438; custom-made by Dako). Beads were pelleted, washed five times in IP buffer with 0.1% Triton X-100, and finally eluted with 40 μl SDS-PAGE sample buffer with DTE. Precipitated proteins were separated by SDS-PAGE and electroblotted onto a PVDF membrane, and phosphorylation was analyzed by autoradiography using the FLA-3000 fluorescence image analyzer (Fujifilm). Precipitated proteins were identified by WB of the same membrane using mouse antisortilin (anti-NTR3, catalog number 612100; BD Biosciences). The degree of sortilin labeling (radioactivity relative to the amount of sortilin precipitated) was evaluated from WBs and autoradiographs using Multi Gauge v.3.2 software.

### *In vitro* kinase assays.

Phosphorylation assays were carried out using 6 μM constitutively active GST-PAK2 (T402E) kinase in a 50-μl volume containing 50 mM Tris-HCl (pH 7.5), 0.1 mM EGTA, 0.1% (vol/vol) 2-mercaptoethanol, 10 mM magnesium acetate, and 0.2 mM [γ-^32^P]ATP (200 cpm/pmol). The following GST fusion proteins (7.5 μM) were added as the substrate: GST, GST–sortilin-cd, GST–sortilin-cd 1–32, GST–sortilin-cd S793D, and GST–sortilin-cd S793A. The phosphorylation assays were carried out at 30°C for 30 min with mild shaking. Reactions were terminated by the addition of 20 μl of 4× NuPAGE LDS sample buffer (Novex) and 5 μl DTE, followed by denaturation at 95°C for 5 min. Samples were separated by SDS-PAGE on NuPAGE 4 to 12% Bis-Tris protein gels, and gels were washed for 10 min in water and fixed according to the manufacturer’s protocol. Phosphorylated products were visualized by autoradiography using the FLA-3000 fluorescence image analyzer.

### Subcellular fractionation.

Subcellular fractionation of transfected HEK cells (HEK/Sortilin Fl and HEK/Sortilin S15D cells) by a discontinuous iodixanol density gradient was performed essentially as described previously by Chang et al. (44). In brief, transfected cells were harvested from two confluent T175 cell culture flasks by centrifugation for 10 min at 3,000 × *g* at 4°C. Cells were homogenized in 3 ml ice-cold solution A (0.25 M sucrose, 1 mM EDTA, and 10 mM HEPES [pH 7.4] plus a cOmplete mini cocktail) and disrupted on ice by 6 passages through a 27-gauge needle, followed by 6 passes through a metal cell cracker with a 9-μm gap. Nuclei and unbroken cells were removed by centrifugation at 1,500 × *g* for 10 min. The postnucleated supernatant was centrifuged for 1 h at 65,000 × *g* at 4°C. The resultant membrane pellets were resuspended in 0.8 ml of solution A. Discontinuous iodixanol density gradients were prepared starting from an OptiPrep gradient stock solution (catalog number D1556; Sigma) containing a final concentration of 50% OptiPrep in 250 mM sucrose, 1 mM EDTA, and 10 mM HEPES (pH 7.4). The resuspended vesicle fractions were loaded on top of the gradients and centrifuged in an SW41 rotor at 40,000 rpm for 2.5 h at 4°C. Twenty-four fractions of 0.5 ml were collected. Fractions (15 μl) were subsequently analyzed by WB using NuPAGE 4 to 12% Bis-Tris gels, nitrocellulose iBlot transfer stacks (Invitrogen), and mouse antisortilin (catalog number 612100).

### Immunocytochemistry.

Cells grown on poly-l-lysine-coated coverslips were fixed for 15 min in 4% paraformaldehyde, washed 3 times with PBS (pH 7.4), and permeabilized using immunocytochemistry (ICC) buffer (0.25% [wt/vol] saponin, 10% [vol/vol] FBS, PBS [pH 7.4]) for 30 min at room temperature. Cells were then incubated in ICC buffer supplemented with primary monoclonal mouse antisortilin (clone F11; produced in-house) overnight at 4°C. Cells were washed 3 times and incubated with Alexa Fluor 488-conjugated donkey anti-mouse antibody (catalog number A21202; Invitrogen) in ICC buffer for 1 h at room temperature. Cells were washed 3 times in PBS, and nuclei were visualized with Hoechst stain (Sigma) and mounted using fluorescence mounting medium (catalog number S3023; Dako). Images were acquired with a Zeiss LSM710 confocal microscope.

### Turnover of metabolically labeled sortilin.

Newly synthesized proteins of HEK293 cells stably transfected with sortilin or sortilin S793D (sortilin S15D) were metabolically labeled with ∼200 μCi/ml [^35^S]Cys-Met (Pro-mix; GE Healthcare) in DMEM without Cys-Met supplemented with 2% (vol/vol) dialyzed FBS and brefeldin A (10 μg/ml; Pierce) for 2 h at 37°C. Following labeling, brefeldin A was washed out, and cells were incubated in DMEM with 2% FBS. At different time points (0, 1, 3, 6, and 18 h), cell lysates were harvested and subjected to immunoprecipitation using anti-sortilin-cd (catalog number 5448; custom-made by Dako). Precipitated proteins were subjected to SDS-PAGE and visualized by phosphorimaging.

### Internalization.

HEK cells expressing either the wild-type or S15D mutated sortilin receptor grown on poly-l-lysine-coated coverslips were incubated for 1 h on ice in DMEM with monoclonal mouse antisortilin clone F11 antibodies. Cells were washed and incubated with warm DMEM. After 0, 5, 10, 20, and 40 min at 37°C, cells were fixed in 4% paraformaldehyde and stained with Alexa Fluor 488-conjugated donkey anti-mouse antibody (as described above for immunocytochemistry), and the degree of receptor endocytosis was evaluated by using a Zeiss LSM710 confocal microscope.

### Mass spectrometry.

The GST-tagged cytoplasmic domain of sortilin (sortilin-cd) (described above) was subjected to *in vitro* phosphorylation by using the constitutively active GST-PAK2 kinase (as described above for the *in vitro* kinase assay). Proteins were subsequently separated by SDS-PAGE and visualized by silver staining. Bands of interest were excised and prepared for in-gel digestion (45), and sequencing-grade porcine trypsin was added (Promega). To evaluate the presence of phosphopeptides in the digests, the material was processed using TiO_2_ microcolumns (46). Peptides were eluted directly onto a stainless steel matrix-assisted laser desorption ionization (MALDI) target using 2,5-dihydroxybenzoic acid as a matrix support and analyzed by using an Autoflex Smartbeam III instrument (Bruker) operated in linear and positive mode. Prior to analyses, the instrument was calibrated by external calibration using a peptide mix containing 7 calibrants (Bruker). The obtained data were evaluated by using GPMAW software.
